# Case Report: An Imported Case of Typhoid Fever Combined with Rhabdomyolysis and Multiple Organ Lesions in China

**DOI:** 10.4269/ajtmh.22-0632

**Published:** 2023-10-09

**Authors:** Qian Huang, Yue Shi, Jingying Xu, Fei Wang, Yinghui Li

**Affiliations:** Department of Infectious Diseases, Affiliated Hangzhou Xixi Hospital, Zhejiang University School of Medicine, Hangzhou, China

## Abstract

Here, we report a case of blood culture-confirmed typhoid fever, rhabdomyolysis, and multiple organ damage that arrived in our country from overseas. A 23-year-old male patient presented at our hospital with fever and muscle pain; the condition progressed rapidly. Six days after the onset of symptoms, the patient developed rhabdomyolysis and liver/kidney damage; levels of creatine kinase (CK; maximum peak: 729,869 U/L) and myoglobin (> 3,000 ng/mL) were extremely high, although the extent of renal damage was relatively mild. Blood culture showed *Salmonella typhi*. The patient received a combination of meropenem and levofloxacin anti-infective therapy, as well as fluid and nutritional metabolic support. He gradually recovered and was discharged after two negative blood cultures. This case highlights the fact that typhoid-induced rhabdomyolysis is a serious, life-threatening disease and that the levels of CK and myoglobin are useful indicators for evaluating typhoid-induced rhabdomyolysis. Clinicians should remain vigilant regarding travel-related illnesses associated with enteric fever.

## INTRODUCTION

Typhoid fever is an acute intestinal infection caused by the bacterium *Salmonella type* that is particularly prevalent in Africa and Southeast Asia.^1^ Typhoid fever can be accompanied by a range of major complications, including hepatitis, intestinal ulcers, and bleeding. Despite the gradual decline in the incidence of typhoid fever in China over recent years, increased international exchange and travel activities have led to imported tropical cases of typhoid fever being detected in clinical practice. Here, we report a case of imported *Salmonella typhi* septicemia with rhabdomyolysis in which the levels of creatine kinase (CK) were extremely high; however, only relatively mild renal damage was evident.

## CASE DESCRIPTION

A 23-year-old man was admitted to our hospital on the September 24, 2018 with a 4-day history of fever, muscle pain, and darkened urine. He had arrived in China from Zambia 6 days earlier. He had no medical history and did not use illicit drugs or smoke tobacco. The patient had a pulse rate of 140 beats per minute, a blood pressure of 143/80 mm Hg, a body temperature of 40°C, a respiratory rate of 40 breaths per minute, and 98% oxygen saturation while breathing room air. Laboratory data were significant for serum total bilirubin (29 µmol/L; normal range: 5–22.1 µmol/L), alanine aminotransferase (398 U/L, normal range: 0–50 U/L), aspartate aminotransferase (1,624 U/L, normal range: 0–50 U/L), glutamyl transpeptidase (76 U/L, normal range: 0–52 U/L), lactate dehydrogenase (38,250 U/L, normal range: 90–250 U/L), CK (> 100,000 U/L, normal range: 24–195 U/L), creatine kinase isoenzyme CK-MB (4,120 U/L, normal range: 0–25 U/L), urea nitrogen (4.0 mmol/L, normal range: 1.7–8.3 mmol/L), creatine ([Cr]; 116 µMol/L, normal range: 53–111 μmol/L), high-sensitivity troponin T (1.28 ng/mL, normal 0–0.014 ng/mL), and myoglobin (> 3,000 ng/mL, normal 28–72 ng/mL). The patient’s test results suggested multiorgan damage to the liver, kidneys, and myocardium, along with rhabdomyolysis. The levels of CK reached a maximum of 729,869 U/L on day 2 after admission, and Cr levels reached a maximum of 138 µMol/L on day 3 after admission and returned to normal levels after the 5th day of admission (Table 1). Lung computed tomography (CT) showed that the texture of the two lungs was still clear, and there were a few cords under the pleura on the dorsal side of the lower lobe of the two lungs. There was no obvious abnormal density shadow in the remaining lungs. Abdominal enhanced CT suggested hepatic cyst. On the fourth day after admission, all four blood cultures (drawn on the day of admission) were positive for *Salmonella typhi*. The strain was resistant to ampicillin, cotrimoxazole, and cefazolin and sensitive to imipenem, ertapenem, ciprofloxacin, levofloxacin, ceftazidime, ceftriaxone, and cefepime. Two stool cultures, taken after admission, were negative. On day 14 of admission, the Widal test revealed *Salmonella typhi* O 1:320 and H antibody 1:320. Electrocardiography was normal. After hospitalization the patient had a high index of infection and was given empirical antimicrobial medication (piperacillin tazobactam 0.5 every 8 hours). At that time, there was no stool, and stool culture was obtained after treatment. This may explain the negative stool culture results. Based on susceptibility results, levofloxacin (0.5 daily), combined with imipenem cilastatin (1.0 every 8 hours), was administered for 2 weeks. The patient received conventional fluid support, nutritional and metabolic support, and other forms of symptomatic treatment. On day 12 of hospitalization, liver and kidney function, along with body temperature, were normal. Furthermore, CK and myoglobin levels had decreased significantly and were almost normal (Table 1). The patient was subsequently discharged after two negative blood cultures.

**Table 1:** Patient’s blood test results and vital signs by date

Test results/vital signs	9/24	9/25	9/26	9/28	9/30	10/3	10/7	10/11	10/15	10/16	10/18
WBC (3.5–9.5 × 10^9^/L)	6.54	6.14	4.63	5.96	14.97	15.01	6.73	6.16	4.50	4.56	3.65
NEUT ratio (40–75%)	79.70	91.20	83.6	73.30	69.20	68.50	52.6	51.40	37.50	39.80	51.50
NEUT count (1.8–6.3 × 10^9^/L)	5.21	5.60	3.87	4.37	10.36	10.28	3.54	3.17	1.69	1.81	1.88
LYM ratio (20–50%)	15.40%	6.20%	11.70%	16.70%	23.30%	24.40%	37.10%	37%	49.10%	50.0%	37%
Eos ratio (0.4–8%)	0%	0%	0%	0%	0.1%	0.1%	0.9%	0.60%	2%	1.8%	1.90%
PLT (120–350 × 10^9^/L)	146	99	105	164	160	144	120	150	140	122	179
HCRP (0–10 mg/L)	133	123	89	40	25	7	5	3	2	–	1
PCT (0–0.5 ng/L)	1.4	1.67	1.620	1.320	0.623	0.269	0.080	0.063	0.051	–	0.036
Temperature (°C)	40	38.8	39.2	39	37.4	36.5	36.3	36.7	36.2	36.3	36.1
HR (beats/minute)	130	108	100	89	86	72	68	72	66	70	65
CK (24–194 U/L)	> 100,000	729,869	699,683	616,407	148,720	7,096	3,142	1,478	1,343	1,764	1,314
CK-MB (0–25 U/L)	4,120	6,016	5,033	3,590	833	114	78	125	154	175	–
LAT (0.1–2.7 mmol/L)	2.7	2.5	2.6	1.9	2.6	2.2	2.2	1.9	1.5	–	1.6
LDH (90–250 µ/L)	38,250	14,209	22,575	14,632	3,221	711	409	365	443	589	–
AST (0–50 U/L)	1,624	1,923	3,591	5,062	3,167	526	140	65	48	62	48
ALT (0–50 U/L)	398	433	723	1,174	1,155	638	226	114	69	87	71
Total bilirubin (5-22.1 µmol/L)	29	20	17.2	17.7	13.2	13.3	12.1	10.4	8.3	10.6	9.2
Direct bilirubin (1–8 µmol/L)	–	11.6	9.7	9.5	6.7	6.5	5.5	4.7	3.4	4.2	3.5
GGT (0–52 U/L)	76	53	48	84	103	87	59	50	39	44	42
Myoglobin (28–72 ng/mL)	–	> 3,000	–	–	–	2,248	836.6	295	–	233.1	–
Cr (53–111 µmol/L)	116	–	138	104	93	82	73	75	81	78	77
BUN (1.7–8.3 µmol/L)	3.9	–	5.1	5.1	6.5	6.2	4.7	4.4	4.4	4.0	2.5

ALT = alanine transaminase; AST = aspartate transaminase; BUN = blood urea nitrogen; CK = creatine kinase; Cr = creatine; CRP = C-reactive protein; Eos = eosinophils; GGT = gamma-glutamyltransferase; HR = heart rate; LAT = lactic acid; LDH = lactate dehydrogenase; LYM = lymphocyte; NEUT = neutrophil; PLT = platelet count; WBC = white blood cell count. – Indicates means no test was done.

## DISCUSSION

Here, we report an imported case of *Salmonella typhi* sepsis with rhabdomyolysis and mild renal injury. The prevalence of typhoid fever is currently high in the Asia-Pacific region^1^; in 2017, the global incidence of typhoid and paratyphoid fever was 0.197%; this compares with 0.05% in East Asia and 0.549% in South Asia.^2^ However, the combination of typhoid with rhabdomyolysis has been reported far less common. Our case had traveled from Zambia to study in China 6 days before disease onset. To our knowledge, no Chinese cases of typhoid fever with rhabdomyolysis have been reported in any of the existing literature. With international exchanges becoming more frequent, cases of travel-related fever are a growing concern.

Rhabdomyolysis is characterized by various causes of muscle damage that lead to the release of cellular components in the blood. Rhabdomyolysis can be triggered by trauma, toxins, rigorous physical exercise, the drug side effects, and genetics.^3^ Less frequent predisposing factors include infections, metabolic disturbance, and excessive heat.^4^ The clinical presentation of rhabdomyolysis varies greatly depending on the primary cause, including myalgia, weakness, and dark urine. Other features include elevated levels of creatine kinase in the blood, severe cardiac arrhythmias, and disseminated intravascular coagulation. The mortality rate of rhabdomyolysis ranges from 8% to 10%.^5^^,^^6^ Acute renal failure (ARF) is the most serious complication of rhabdomyolysis and is reported to occur in 14% to 46% of cases, mostly due to tubular necrosis caused by myoglobin blockage.^7^^,^^8^ ARF is closely associated with the prognosis of rhabdomyolysis. A previous study reported a mortality rate of 42% in an ARF group of patients; this compared with 8% in a group of patients without ARF.^9^ Some studies have reported that CK levels can predict renal injury complicated by rhabdomyolysis.^10^^,^^11^ Previous literature reported that renal injury was more prominent in cases of rhabdomyolysis induced by typhoidal and nontyphoidal *Salmonella*.^12^^,^^13^ In our present case, there was evidence of multiorgan damage to the liver, myocardium, and rhabdomyolysis. The elevated levels of CK (maximum peak: 729,869 U/L) and myoglobin (> 3,000 ng/mL) were particularly pronounced compared with previously reported cases of rhabdomyolysis induced by nontyphoidal *Salmonella*,^14^ although the renal injury in our case was not as prominent. This might suggest that elevated levels of CK may be more reflective of the degree of damage to the transverse muscle in addition to being associated with renal injury. On the other hand, a previous multicenter retrospective study indicated that serum phosphate and potassium levels at admission, as well as myoglobin levels > 8,000 U/L were predictive of stage 2 to 3 acute kidney injury.^8^ Nevertheless, CK plays a key role in the diagnosis and assessment of rhabdomyolysis.^15^ Similar to nontyphoidal rhabdomyolysis, typhoidal rhabdomyolysis can be complicated by multiple organ injuries, and rhabdomyolysis is more likely to occur in bacterial sepsis including *Salmonella typhi*.^12^^,^^16^ Further clinical validation is needed in the future.

If left untreated, most patients will develop complications by the 3rd week after the onset of symptoms.^1^^,^^17^ In our patient, the disease progressed rapidly and examinations for admission suggested multi-organ damage. *Salmonella enterica *serovar type can cause a life-threatening and systemic infection known as typhoid fever. Previous studies have indicated that typhoid toxin is likely to play a central role in the pathogenesis of typhoid fever.^18^^,^^19^ Many genes (phosphofructokinase, phosphoglycerate mutase 2, carnitine palmitoyltransferase II) encoding the proteins involved in sugar and fatty acid metabolism are associated with rhabdomyolysis and are known to participate in the development of rhabdomyolysis.^20^ Another previous study reported that muscular LMNA (A-type lamin) interacting protein causes recessive myopathy with rhabdomyolysis, myalgia, and baseline elevated serum creatine kinase.^21^ Although the expression and function of the genes mentioned here were not tested in our present case, the exact role of these genes in the pathogenesis of rhabdomyolysis needs to be further investigated in the future.

## CONCLUSION

Rhabdomyolysis induced by *Salmonella enterica serovar Typhi* is a clinically rare disease but can be life-threatening. Clinicians need to be aware of this condition, especially with regard to travel-related enteric fever. Levels of CK and myoglobin represent important clinical tests and can play a significant role in the assessment of rhabdomyolysis.
